# Supplementary material for: 5-Iodo-4-thio-2′-Deoxyuridine as a Sensitizer of X-ray Induced Cancer Cell Killing
Source: Int J Mol Sci. 2019 Mar 15;20(6):1308. doi: 10.3390/ijms20061308 (PMC6470520; doi:10.3390/ijms20061308)
Supplement: Supplementary file 1 [file ijms-20-01308-s001.pdf]

Supplementary Materials

5-Iodo-4-thio-2'-deoxyuridine as a Sensitizer of X-ray Induced Cancer Cell Killing

Samanta Makurat, Paulina Spisz, Witold Kozak, Janusz Rak, Magdalena Zdrowowicz*

Faculty of Chemistry, University of Gdańsk, Wita Stwosza 63, 80-308 Gdańsk, Poland

*magdalena.zdrowowicz@ug.edu.pl

Table of Content

Identification of radiolysis products (Fig. S1-S5)…………………..…………………......S3

Transition state structures (Fig. S6-S10)……..…………………………...………………S8

Additional HPLC analyses of the irradiated ISdU (Fig. S11, S12)...……………..,……S13

Clonogenic assay (Fig. S13, Table S1)……………………………………………....S15

Synthesis of the studied analog (Scheme S1).........................…………………...……S16

NMR spectra of the obtained analog (Fig. S14, S15)….......……...……………...……S17

Mass spectra of the obtained analog (Fig. S16, S17)...…..…………......………..……S18

Cytometric analysis of histone H2A.X phosphorylation (Fig. S18)………………...….S21

Cytometric analysis of cell death (Figs S19 and S20)…………………..………...….S22

Identification of radiolysis products

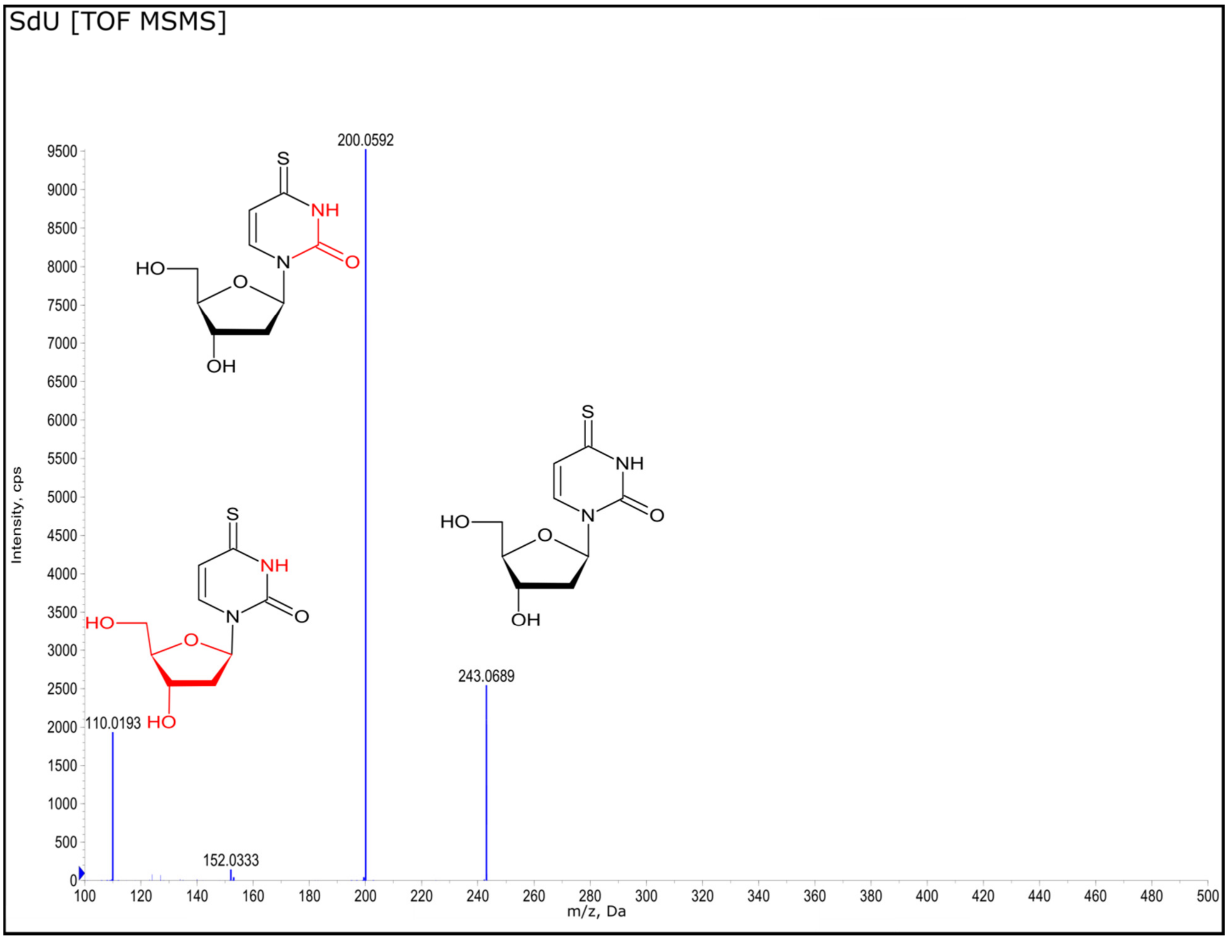

Figure S1. MS/MS spectrum (in negative ionization mode) of SdU and ion identities.

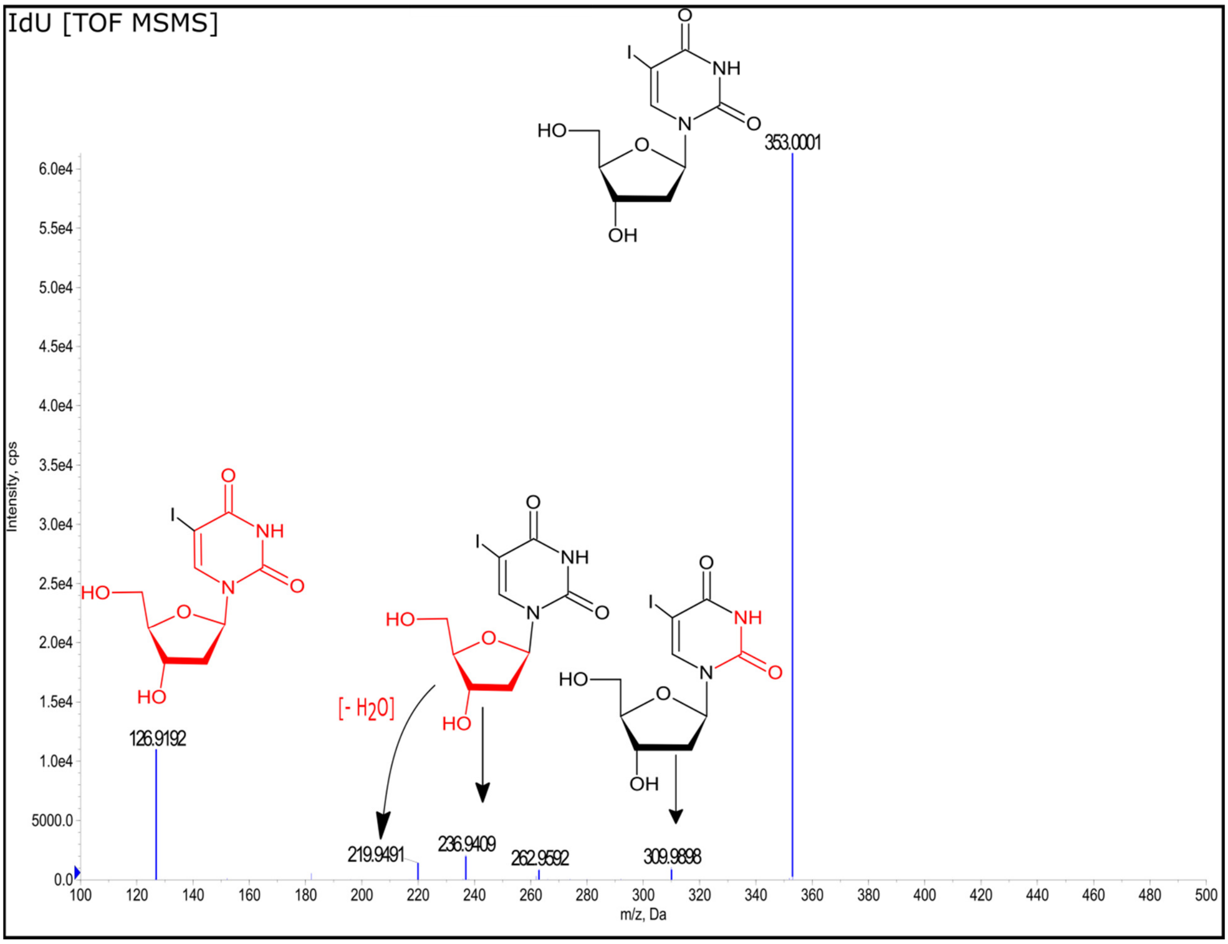

Figure S2. MS/MS spectrum (in negative ionization mode) of IdU and ion identities.

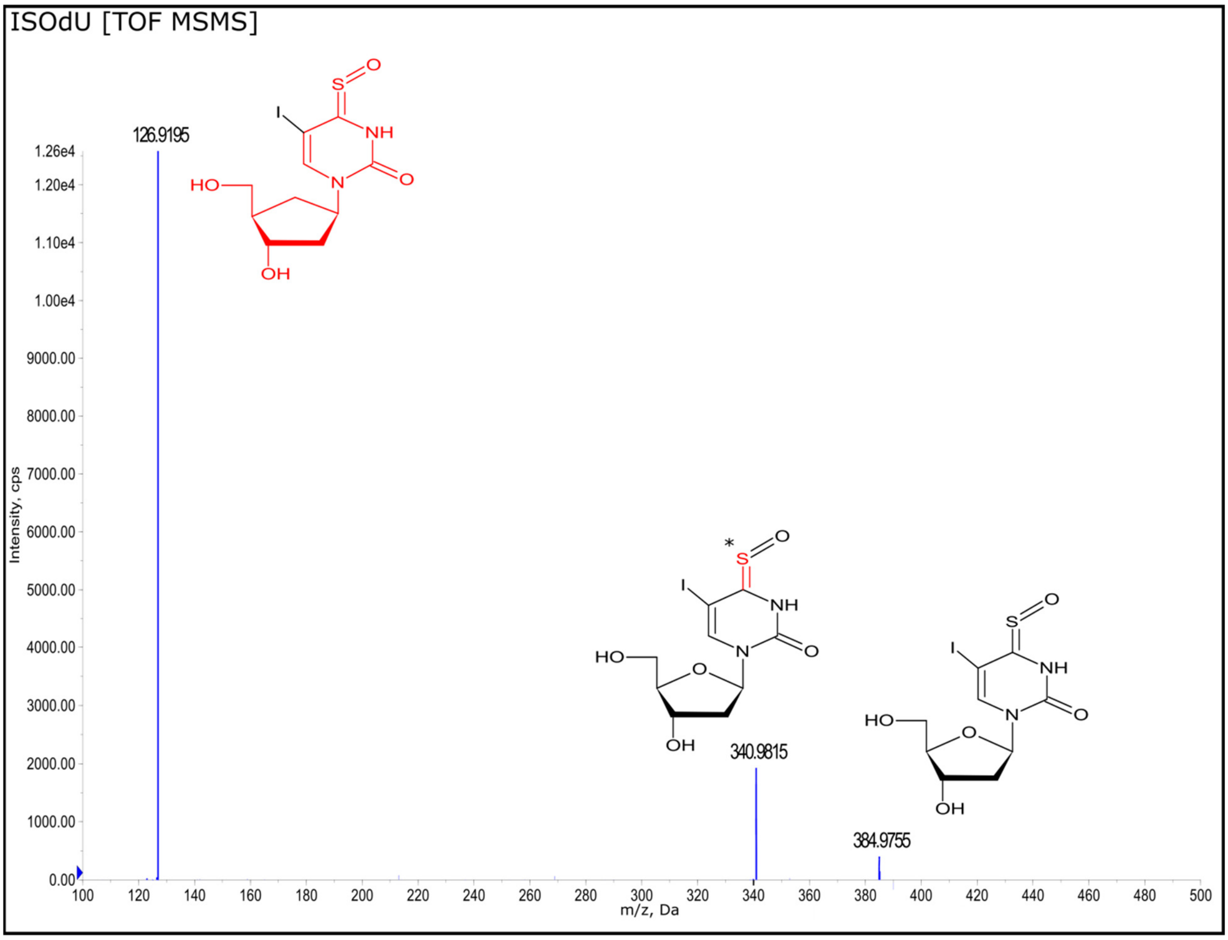

Figure S3. MS/MS spectrum (in negative ionization mode) of ISOdU and ion identities. *Bergman, F., Rahat, M., and Frank, A. (1982) Comparsion of the Mass Spectra of 6-Thiotheophyllines and 6-Sulfinyltheophyllines. *Organic Mass Spectrometry 17*, 565–568.

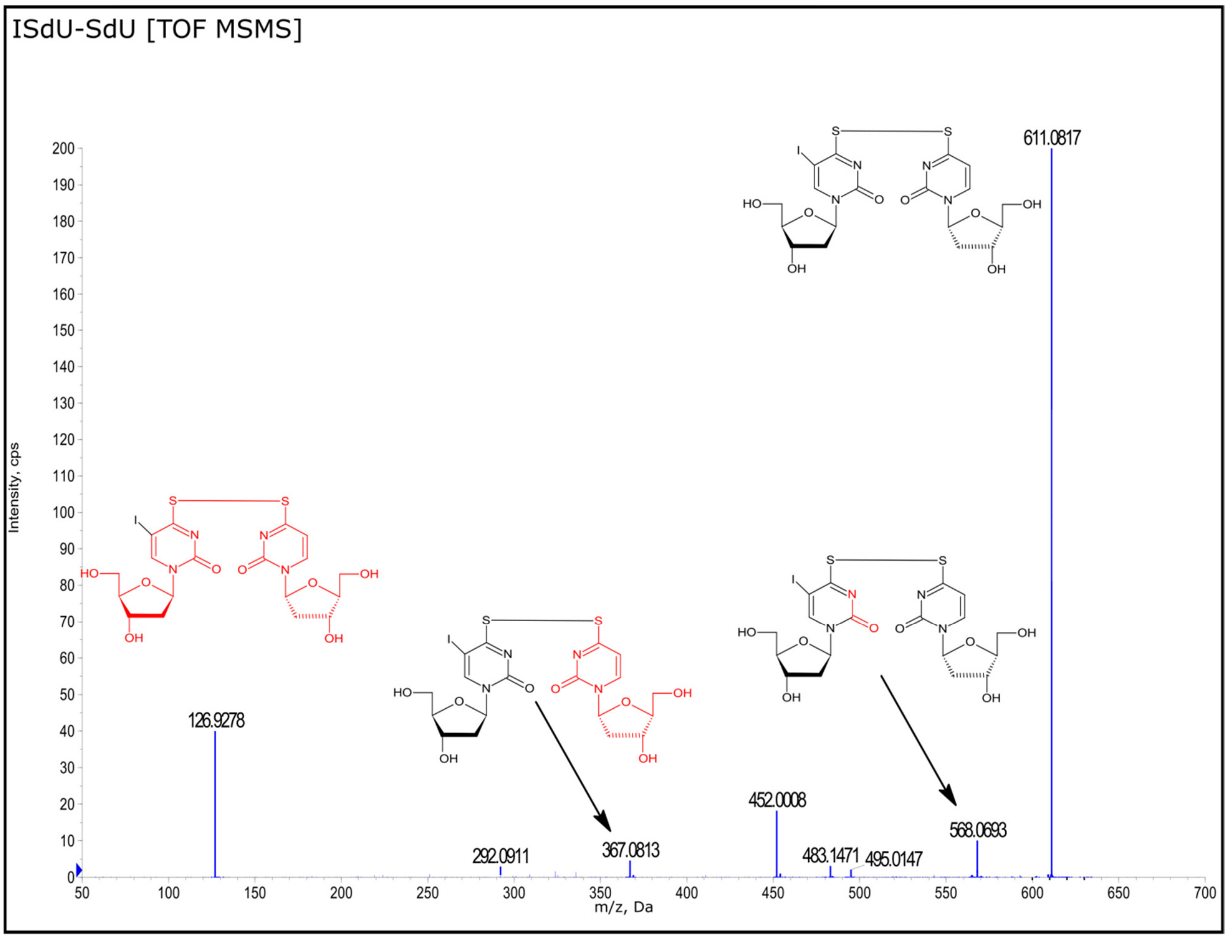

Figure S4. MS/MS spectrum (in negative ionization mode) of dimer ISdU-SdU and ion identities.

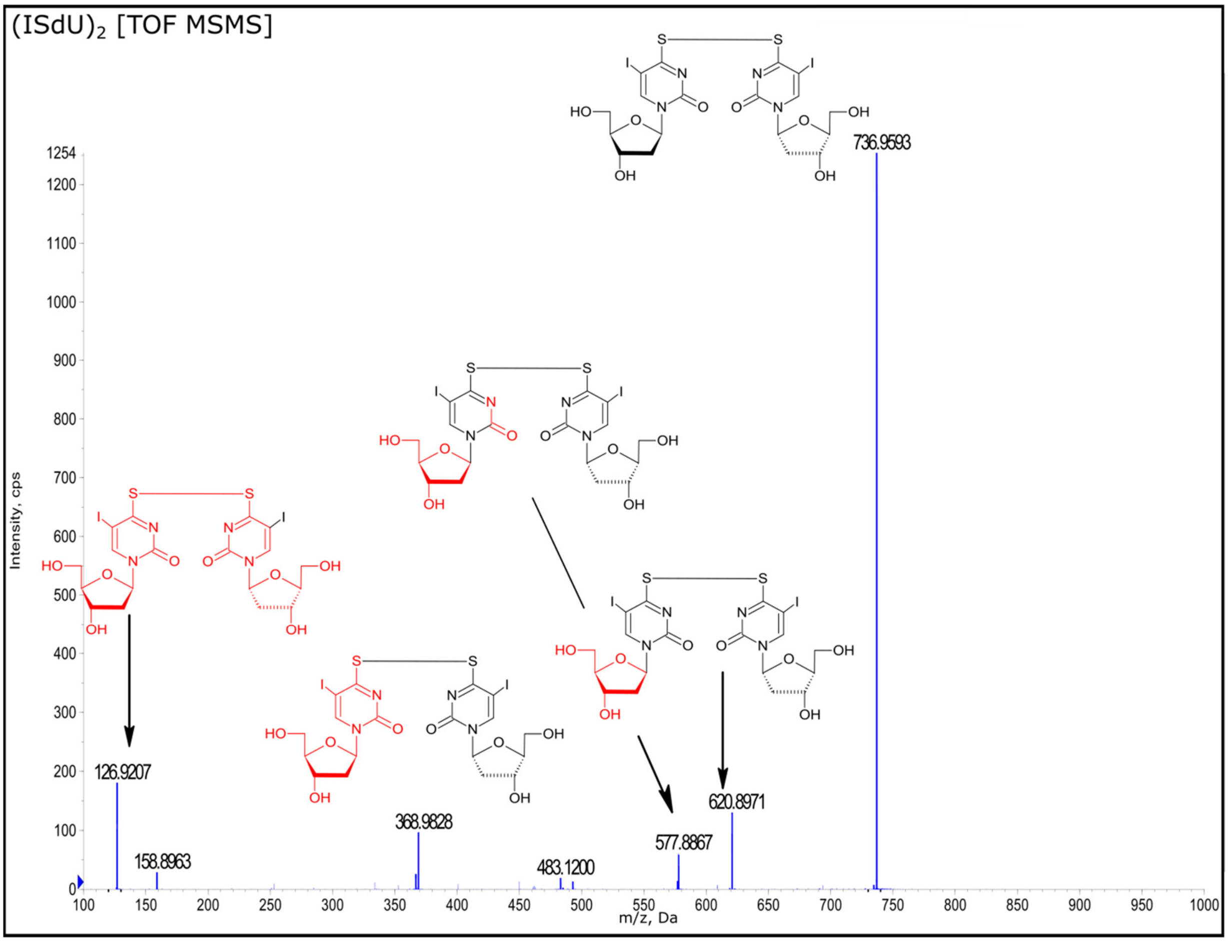

Figure S5. MS/MS spectrum (in negative ionization mode) of dimer $(ISdU)_2$ and ion identities.

Transition state structures

C	0.013906	0.398179	0.051251
N	0.021776	0.004232	1.375463
C	1.240801	-0.142343	2.054473
C	2.403247	-0.292014	1.355578
C	2.431042	-0.157289	-0.041996
N	1.269460	0.490216	-0.531924
I	4.301213	-0.868426	2.609843
S	3.633729	-0.630230	-1.171947
O	-1.019014	0.669179	-0.579790
H	1.182742	-0.091779	3.138003
H	1.256007	0.737044	-1.517335
C	-1.242847	-0.196448	2.085252
H	-2.036891	0.327943	1.555879
H	-1.490539	-1.261515	2.146464
H	-1.154567	0.206886	3.097584

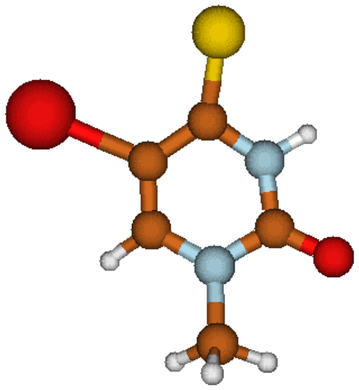

Figure S6. Transition state structure for the DEA process (ΔG* = 2.8 kcal/mol).

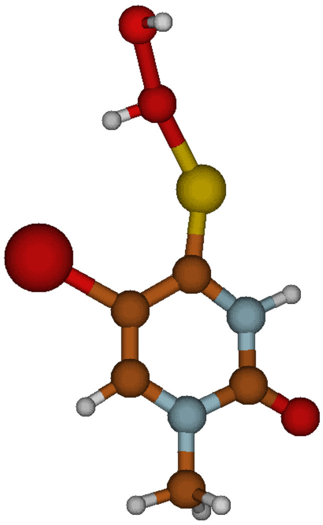

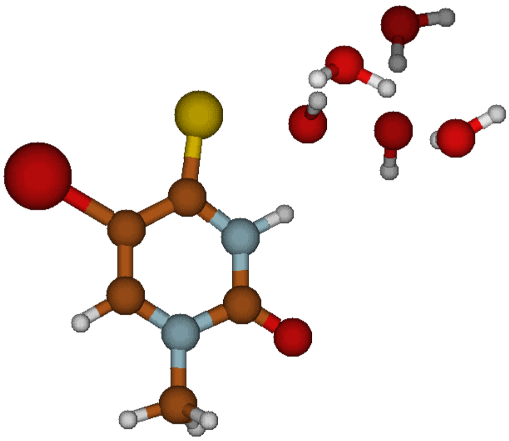

C	-0.177704	0.452238	0.175237
C	-0.066454	0.601291	1.584518
N	1.185103	0.374401	2.102175
C	2.339199	0.027309	1.411625
N	2.152505	-0.120705	0.034331
C	0.944314	0.090055	-0.540341
S	-1.300548	1.046809	2.686815
O	-2.671637	-0.421346	2.395822
O	-4.102440	-1.796522	2.183571
O	3.415837	-0.143278	1.964162
C	3.333296	-0.511414	-0.759480
I	-1.960091	0.853465	-0.883316
H	0.912874	-0.029159	-1.617373
H	1.303771	0.446358	3.111725
H	4.110690	0.245856	-0.648251
H	3.707310	-1.471760	-0.401764
H	3.039005	-0.593103	-1.804171
H	-3.432854	-2.481256	2.010026
H	-3.286658	0.019723	1.779472

S	-0.195294	-0.130700	-0.131170
O	-0.295814	-0.249290	2.156775
H	0.702488	-0.303564	2.188595
H	2.904468	0.310923	1.786463
O	2.478466	-0.296742	2.413025
H	2.514405	0.151943	3.294316
O	1.917610	0.821645	4.866165
H	2.254284	0.339852	5.639317
H	1.015293	0.446172	4.680356
O	-0.371543	-0.343576	3.953532
H	-1.202432	0.157227	4.044070
H	-0.844398	-3.287130	5.047186
O	-0.972410	-3.049328	4.114979
H	-0.760639	-2.083252	4.063276
C	-0.804840	1.451766	-0.198852
C	-1.135254	2.167822	-1.390814
N	-1.012350	2.132995	0.969188
C	-1.616000	3.447995	-1.277122
N	-1.795181	4.067479	-0.079227
C	-1.498467	3.421640	1.121593
C	-2.318716	5.442691	0.008861
H	-2.481319	5.818067	-0.999936
H	-3.260390	5.441100	0.560180
H	-1.596378	6.075031	0.526950
O	-1.653546	3.945873	2.217877
H	-0.790522	1.608105	1.824381
I	-0.892562	1.302793	-3.301597
H	-1.878253	4.034876	-2.150154

Figure S7. Transition state structure for the ISU to ISOU oxidation reaction (for the reaction without (left) and with (right) explicitly added waters ΔG* = 25.5 and 15.3 kcal/mol, respectively).

C	-0.036982	0.029281	0.069061
N	0.004870	-0.043271	1.459725
C	1.128454	-0.050271	2.247506
C	2.349607	0.330873	1.578077
C	2.319038	0.476272	0.223651
N	1.185369	0.291614	-0.527364
S	0.924218	-0.305818	3.961033
O	1.385441	-1.706498	3.084702
I	4.159741	0.628165	2.623467
C	1.205888	0.425862	-1.994595
O	-1.084693	-0.095877	-0.555826
O	-0.394442	-3.707101	2.752730
O	-1.675957	-4.188626	5.183775
O	-0.455496	-2.674609	7.210660
O	1.018355	-5.921813	1.661731
H	0.229560	-2.932324	2.863139
H	-1.002188	-1.879493	7.313749
H	-0.890557	-3.196958	6.495699
H	-1.580265	-5.137254	5.369235
H	-1.219099	-4.040753	4.319602
H	-1.054560	-3.419063	2.099387
H	1.528041	-6.301499	2.395399
H	0.539103	-5.156467	2.052001
H	2.231026	0.609320	-2.312125
H	0.841180	-0.495135	-2.451807
H	0.570047	1.258631	-2.300497
H	-0.897387	-0.192997	1.905304
H	3.203926	0.751792	-0.337982

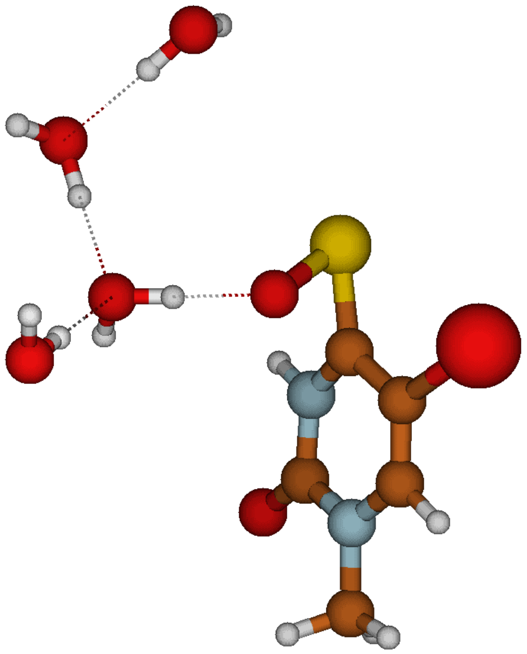

Figure S8. Transition state structure for the ISOU to oxathiirane reaction (ΔG* = 24.5 kcal/mol).

N	0.038951	-0.120731	0.249797
C	0.194774	0.120275	1.612533
N	1.520560	0.200691	2.027281
C	2.614343	0.051298	1.243821
C	2.407419	-0.182585	-0.145707
C	1.112114	-0.258553	-0.581840
O	-0.744048	0.253458	2.384815
O	3.801190	0.134695	1.750072
S	4.025159	0.426863	3.511642
S	1.754433	-2.138802	6.719824
O	1.374837	-3.851227	6.311803
C	1.483633	-4.280293	5.097038
N	1.890225	-3.450859	4.105120
C	2.033547	-3.788929	2.761212
N	1.731719	-5.117492	2.471438
C	1.325315	-5.988416	3.439076
C	1.184681	-5.629755	4.753166
O	2.392420	-2.982242	1.915963
I	0.555397	-7.000814	6.223421
C	1.865835	-5.538407	1.063552
I	4.040798	-0.397649	-1.457026
C	-1.343164	-0.209981	-0.258519
H	1.621628	-6.596895	0.991991
H	1.182394	-4.957853	0.442065
H	2.891053	-5.371725	0.730356
H	2.108450	-2.484031	4.389454
H	1.116158	-6.996941	3.102060
H	-1.307585	-0.414925	-1.326987
H	-1.869563	-1.015599	0.255112
H	-1.860735	0.733838	-0.079697
H	1.712294	0.365578	3.025904
H	0.873900	-0.436143	-1.624162
H	6.875719	-3.639530	2.126735
H	3.779932	-2.149243	1.350127

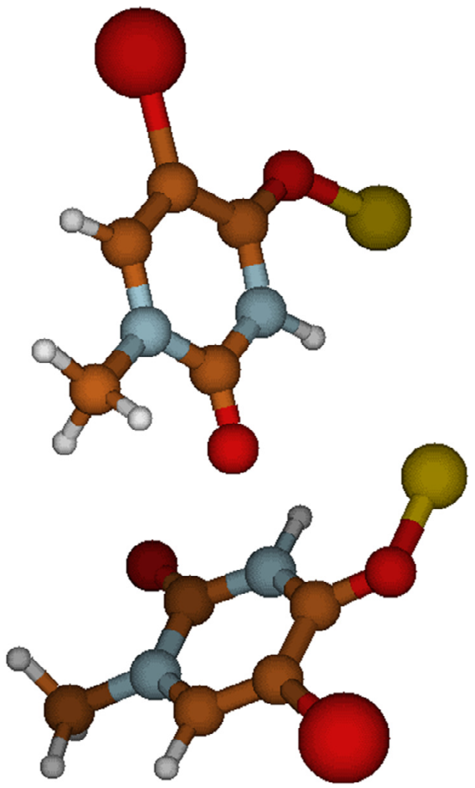

Figure S9. Transition state structure for the sulfur extrusion to form the final IU product (ΔG^* = 1.8 kcal/mol).

C	0.704828	-0.090491	0.129023
C	0.730519	-0.640752	1.386166
C	1.887102	-1.387896	1.760062
N	2.885736	-1.505424	0.859181
C	2.883402	-0.966279	-0.404719
N	1.731275	-0.237303	-0.747661
I	-0.897354	-0.374571	2.705987
S	2.109598	-2.155362	3.283585
O	3.814874	-1.100422	-1.198451
C	1.681920	0.357082	-2.093756
O	4.427568	-2.727158	2.308867
C	4.744001	-4.117361	2.193048
C	3.711148	-4.861877	1.329693
C	6.136720	-4.172919	1.520683
C	4.818005	-4.724656	3.602953
H	-0.140257	0.489131	-0.225291
H	2.514461	1.051345	-2.218898
H	1.756259	-0.431169	-2.844895
H	0.737768	0.887739	-2.208004
H	5.142385	-5.770343	3.560955
H	5.528855	-4.166392	4.221227
H	3.836332	-4.687142	4.086363
H	3.970129	-5.923138	1.248082
H	2.713697	-4.786573	1.774593
H	3.671642	-4.446117	0.316993
H	6.461171	-5.214057	1.411738
H	6.103741	-3.714775	0.526948

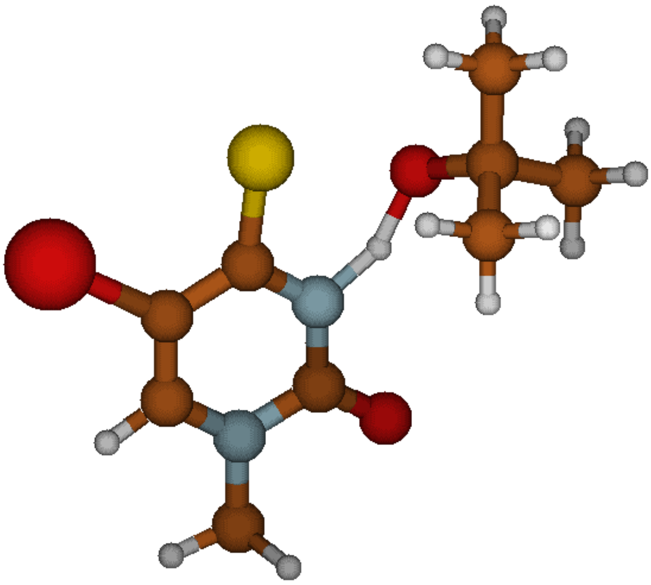

Figure S10. Transition state structure for the reaction of the *t*-BuO$^{\bullet}$ radical with ISU (ΔG^{*} = 0.6 kcal/mol).

Additional HPLC analyses of irradiated ISdU

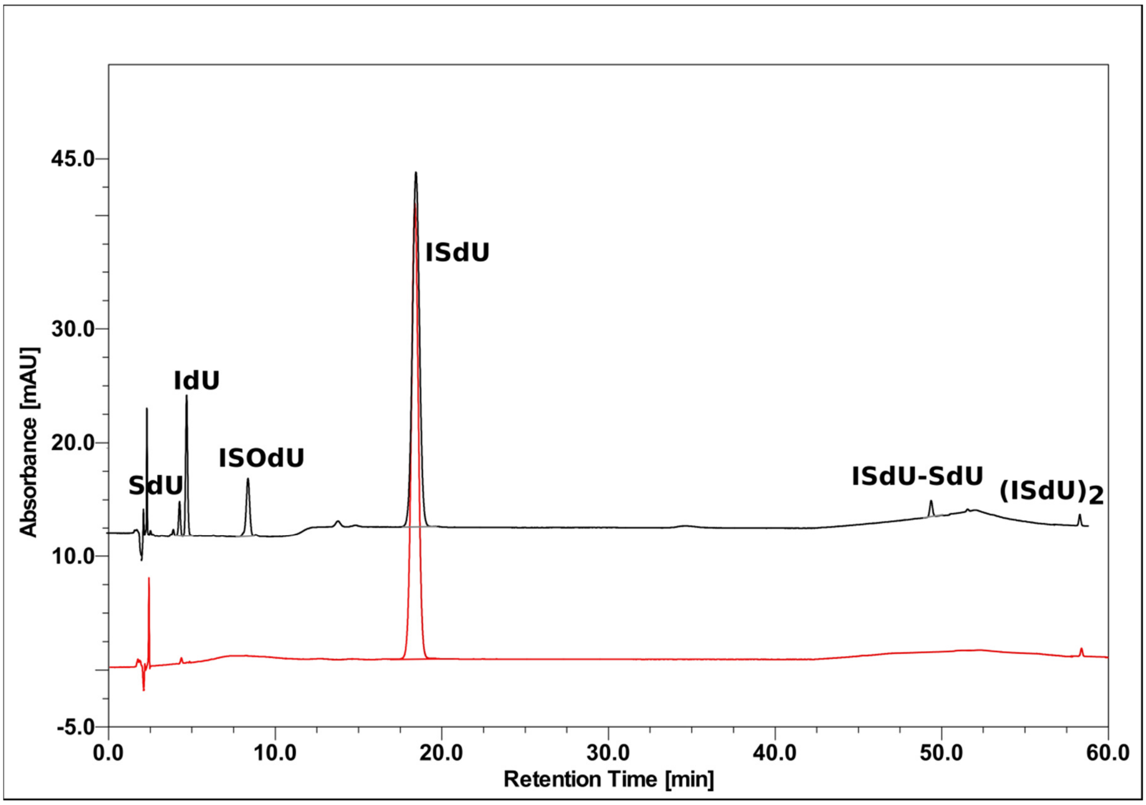

Figure S11. HPLC traces for irradiated (black) and non-irradiated (red) ISdU solution without *t*-BuOH in aerobic conditions.

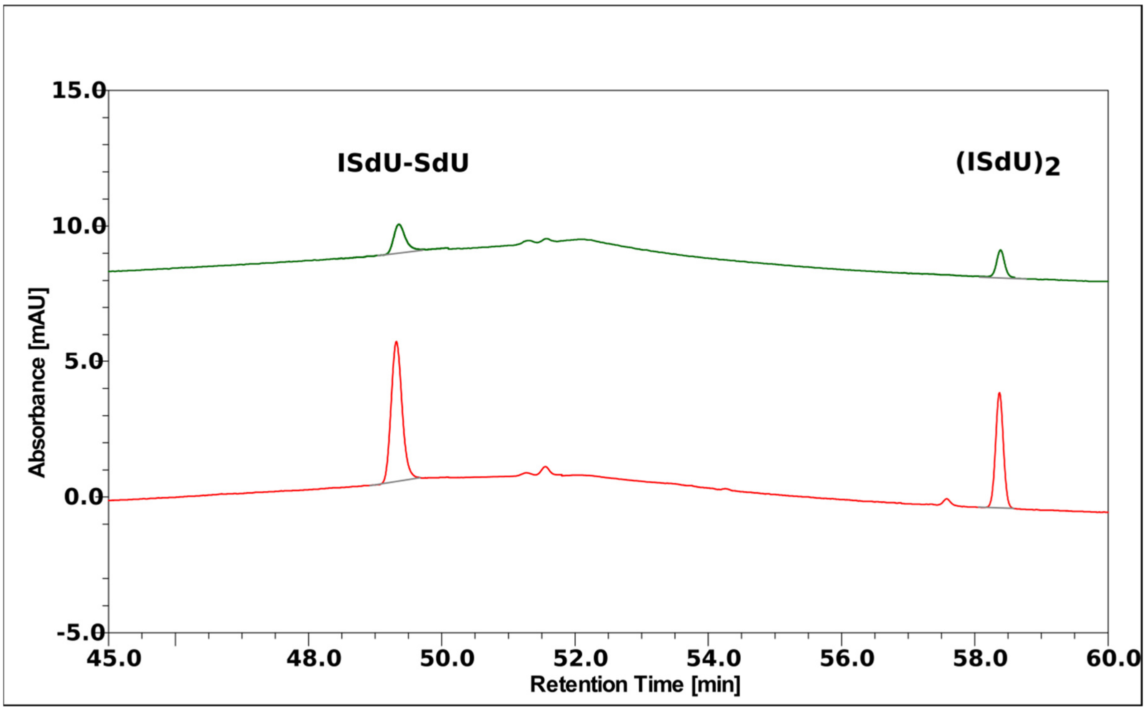

Figure S12. HPLC traces for the dimers formed in aerobic (green) and anaerobic (red) conditions.

Clonogenic assay

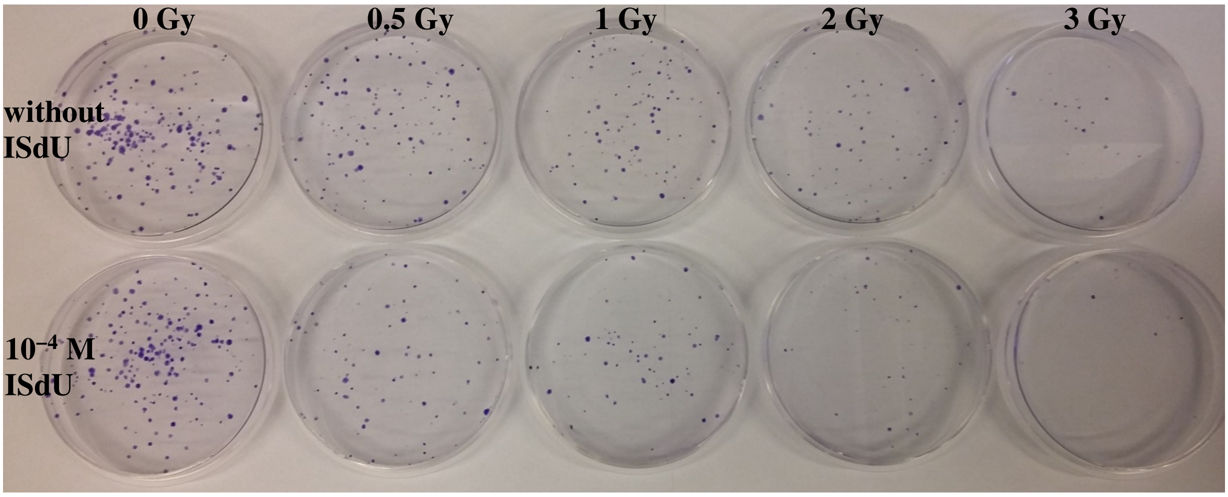

Figure S13. Stained colonies obtained from clonogenic assay.

Table S1. Plating efficiencies [%] for the MCF-7 cells obtained from clonogenic assay.

Dose [Gy]	Plating efficiencies [%]		
	0 mM ISdU	10 mM ISdU	100 mM ISdU
0	26.31±0.09	23.81±0.09	21.31±0.09
0.5	20.63±0.18	16.13	12.75±0.35
1	17.94±0.27	13.06±0.09	8.69±0.27
2	9.75±0.18	6.56±0.27	4.31±0.27
3	4.81±0.27	2.81±0.27	1.44±0.09

Synthesis of the studied analog

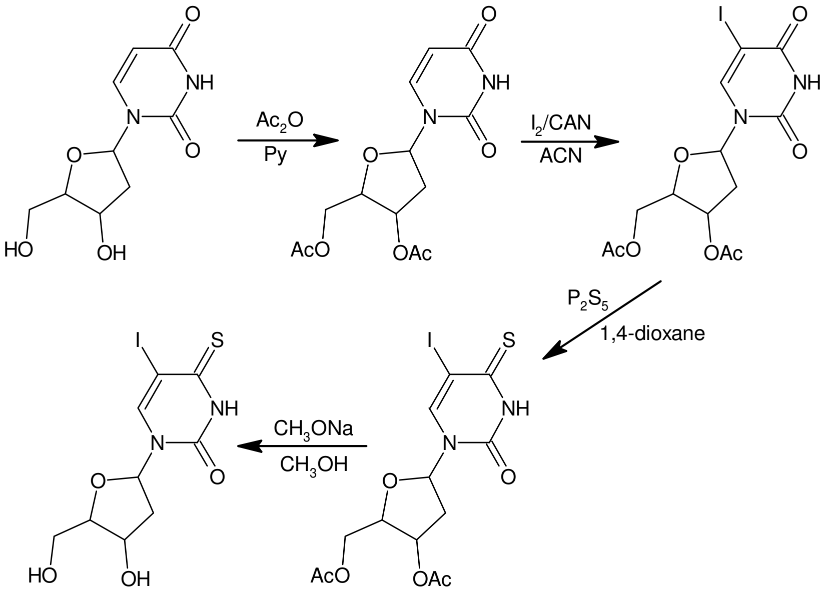

Scheme S1. Synthetic route for ISdU.

NMR spectra of the studied analog

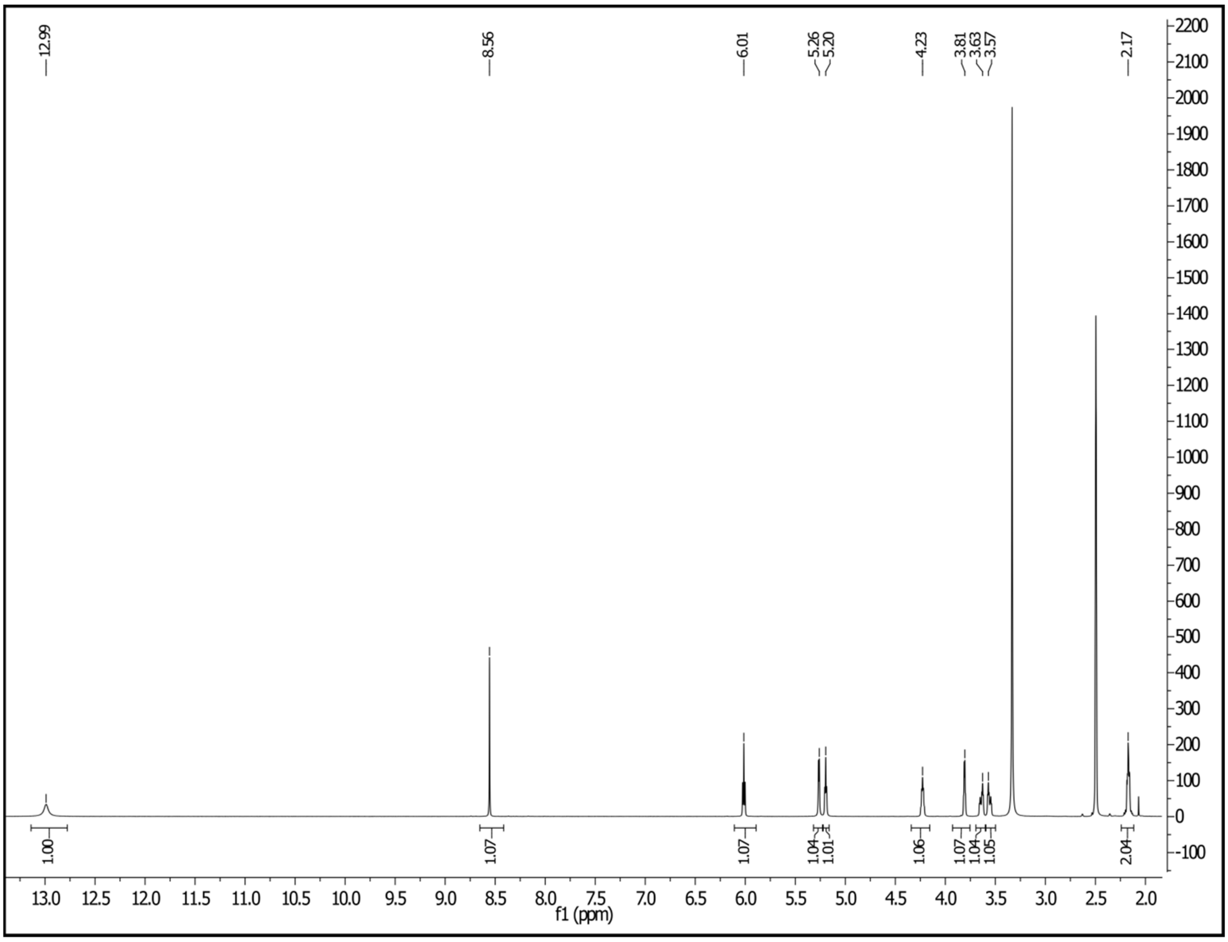

Figure S14. ^{1}H NMR spectrum of ISdU.

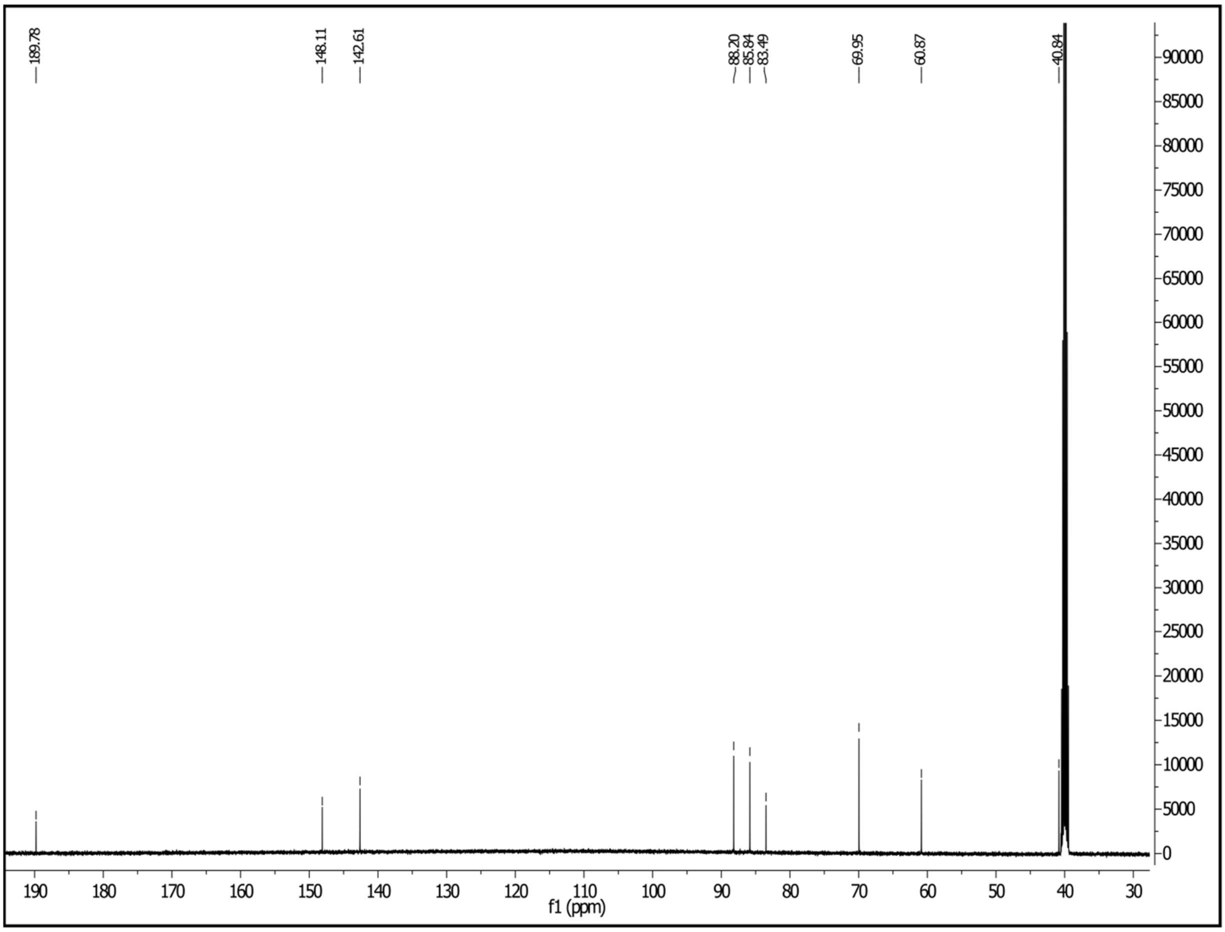

Figure S15. ^{13}C NMR spectrum of ISdU.

Mass spectra of the obtained analog

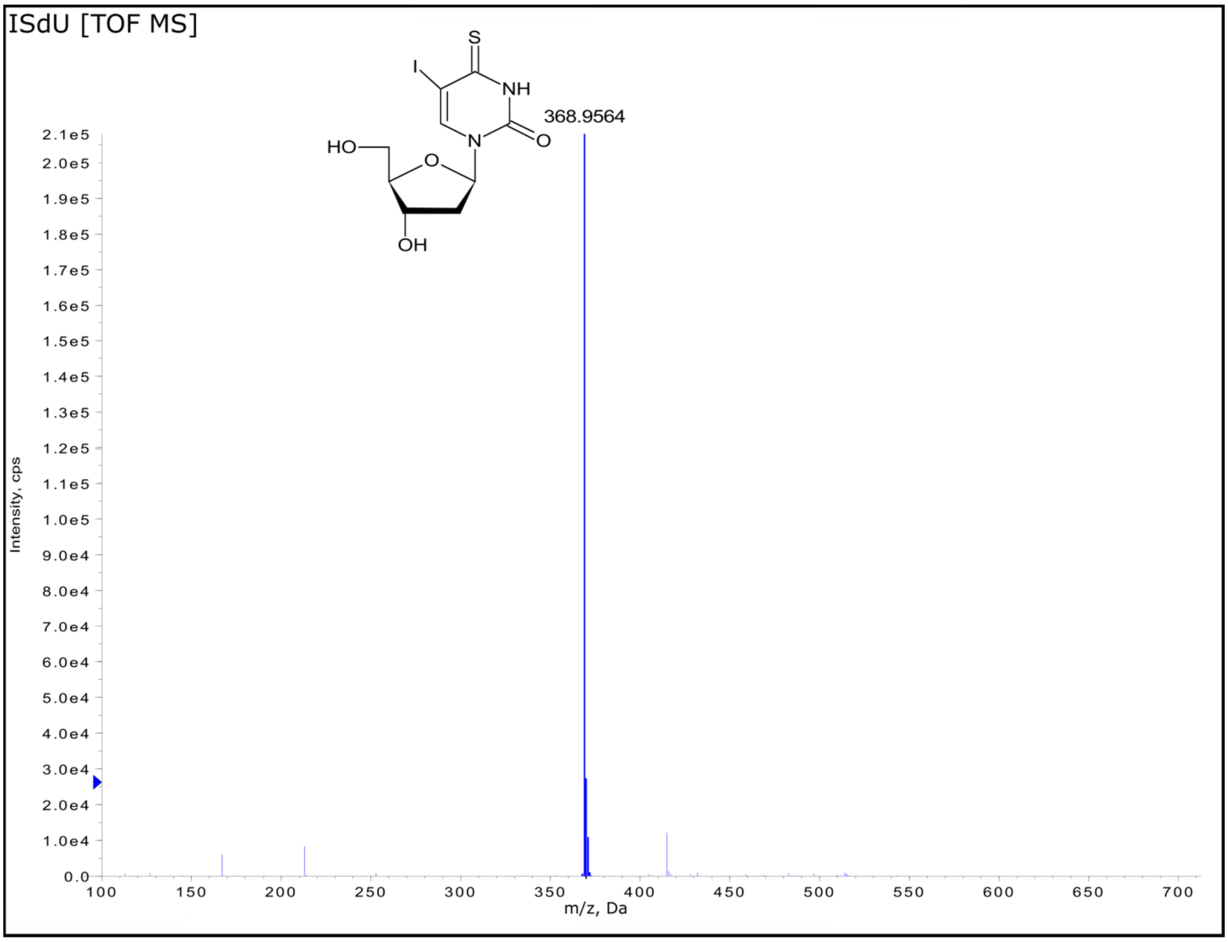

Figure S16. MS spectrum (in negative ionization mode) of ISdU.

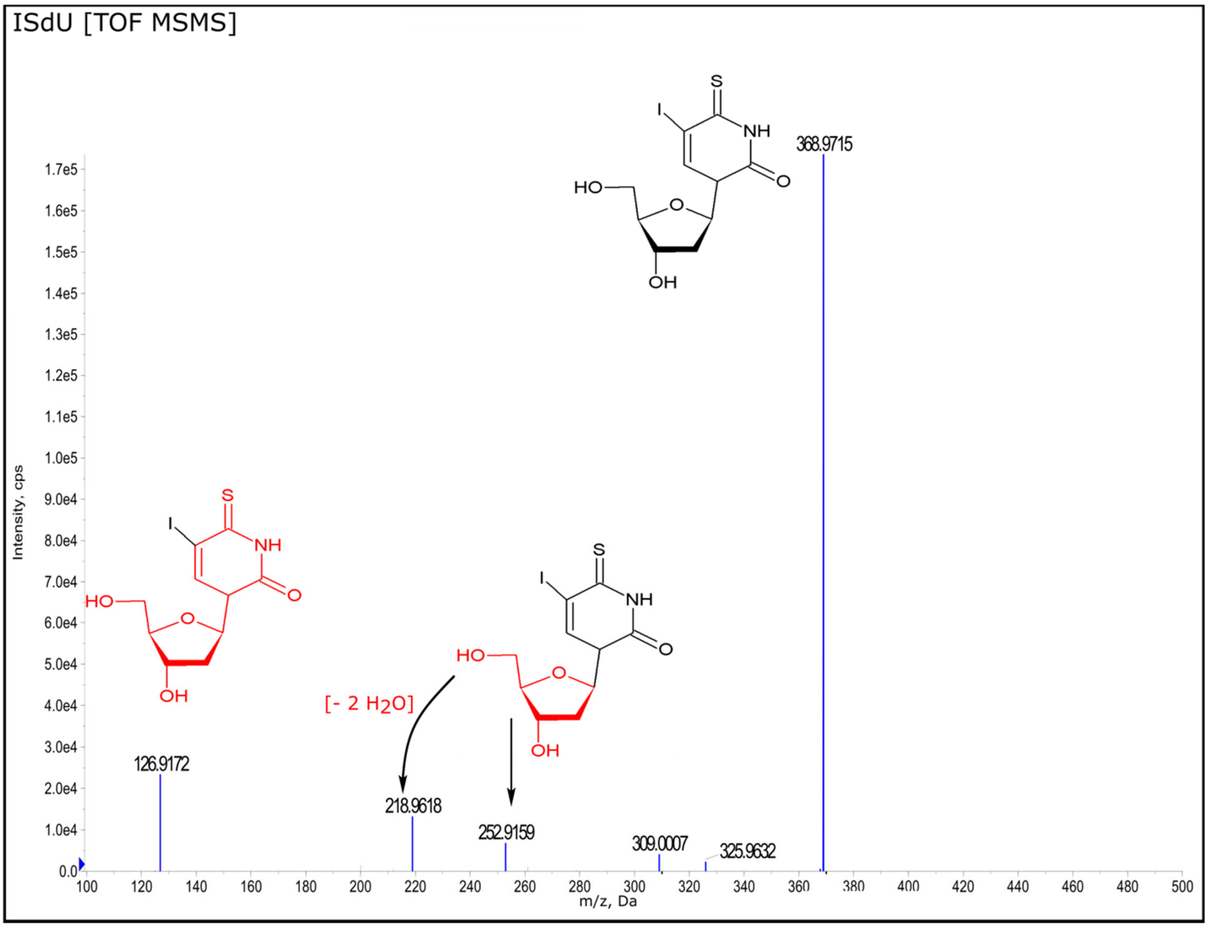

Figure S17. MS/MS spectrum (in negative ionization mode) of ISdU and ion identities.

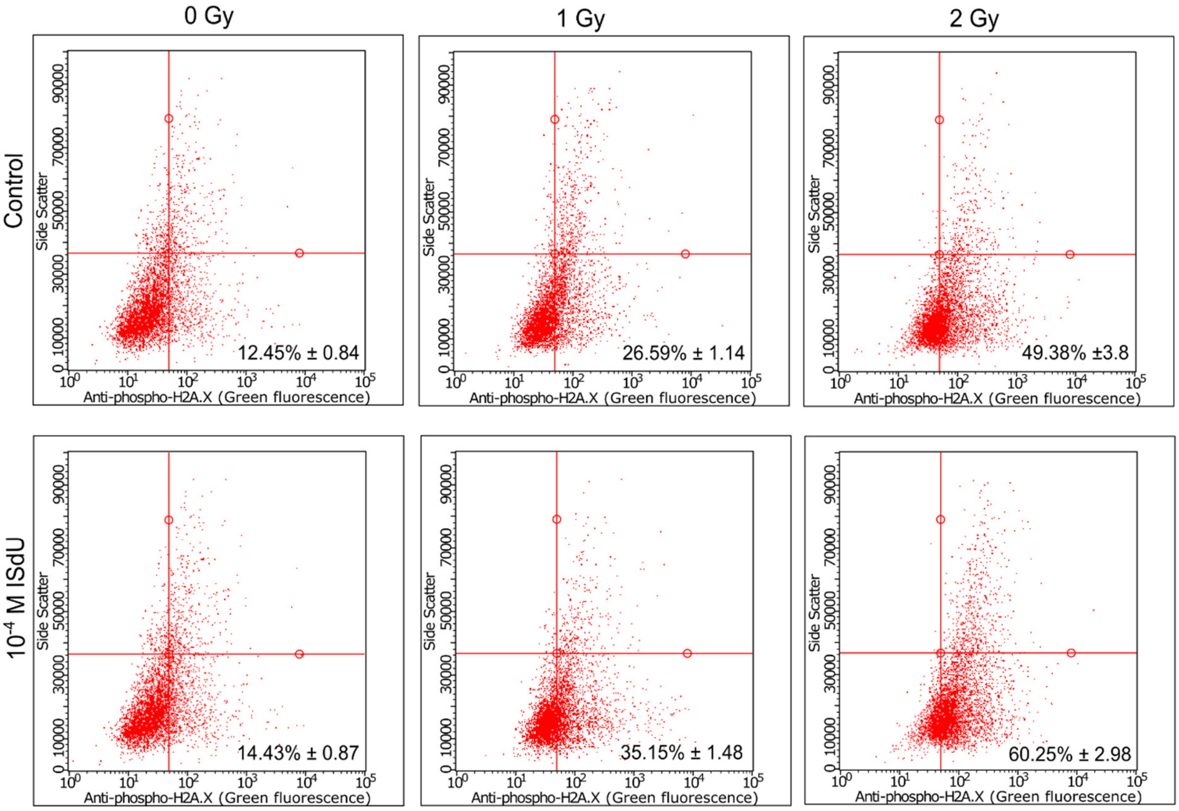

Figure S18. Cytometric analysis of histone H2A.X phosphorylation.

Cytometric analysis of cell death

MCF-7 cells were grown in RPMI supplemented with 10% FBS and antibiotics at a concentration of 100 $U \cdot mL^{-1}$. Cells were treated with ISdU at a concentration of 10^{-4} and incubated (37°C, 5% CO_2) for 48 h. After this time, the plates with cells were irradiated (Cellrad X-ray cabinet, Faxitron X-ray Corporation) with the dose of 2 Gy and incubated for 1 h. Then, the cells were dissociated with Accutase solution, stained and analyzed by flow cytometry (Guava easyCyte™) according to the manufacturer's protocol (FlowCollect™ MitoDamage Kit, Merck).

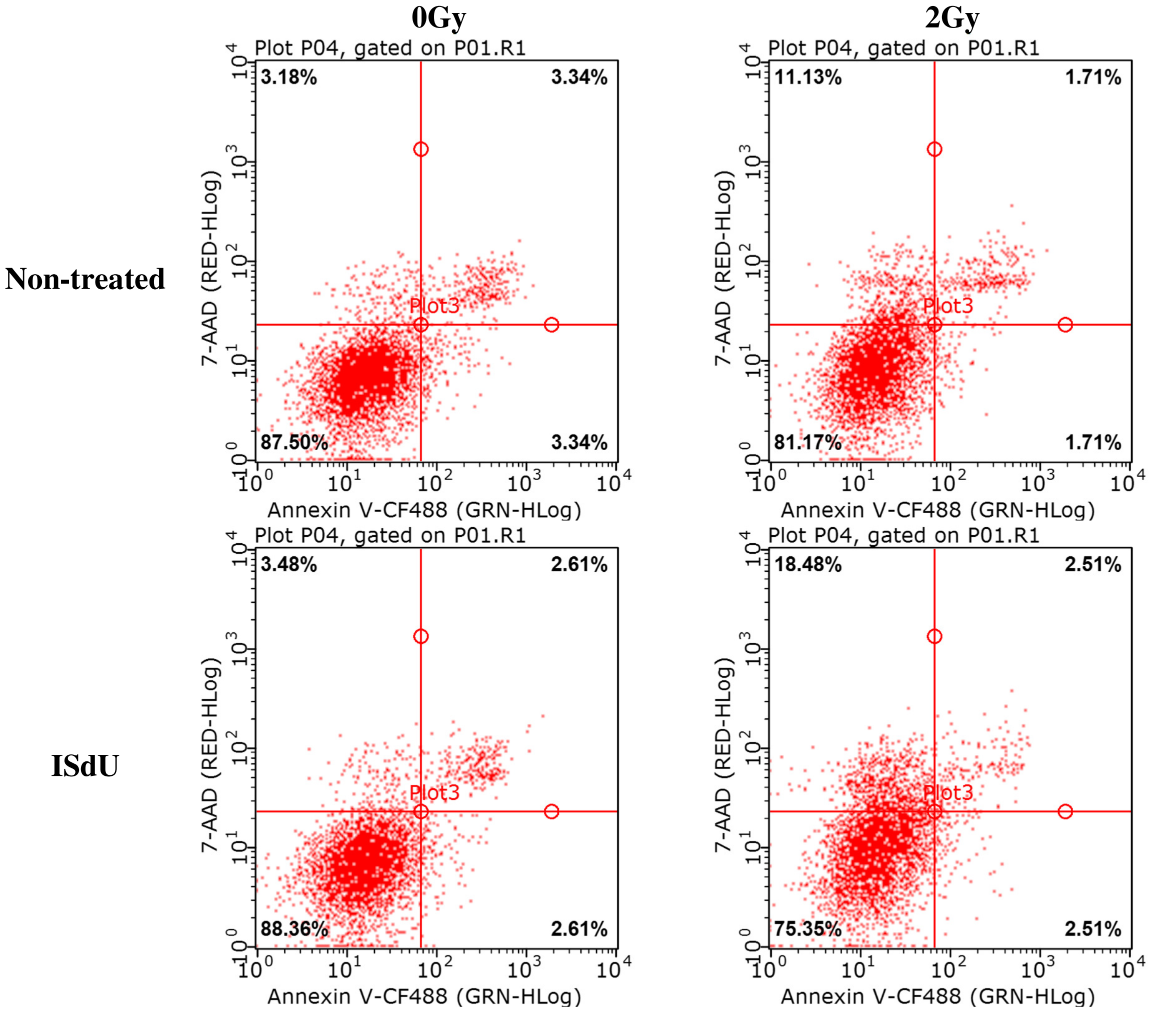

Figure S19. Cytometric analysis of cell death – dot plots provide comparison of 7-AAD (cell death) vs. Annexin V (late apoptosis).

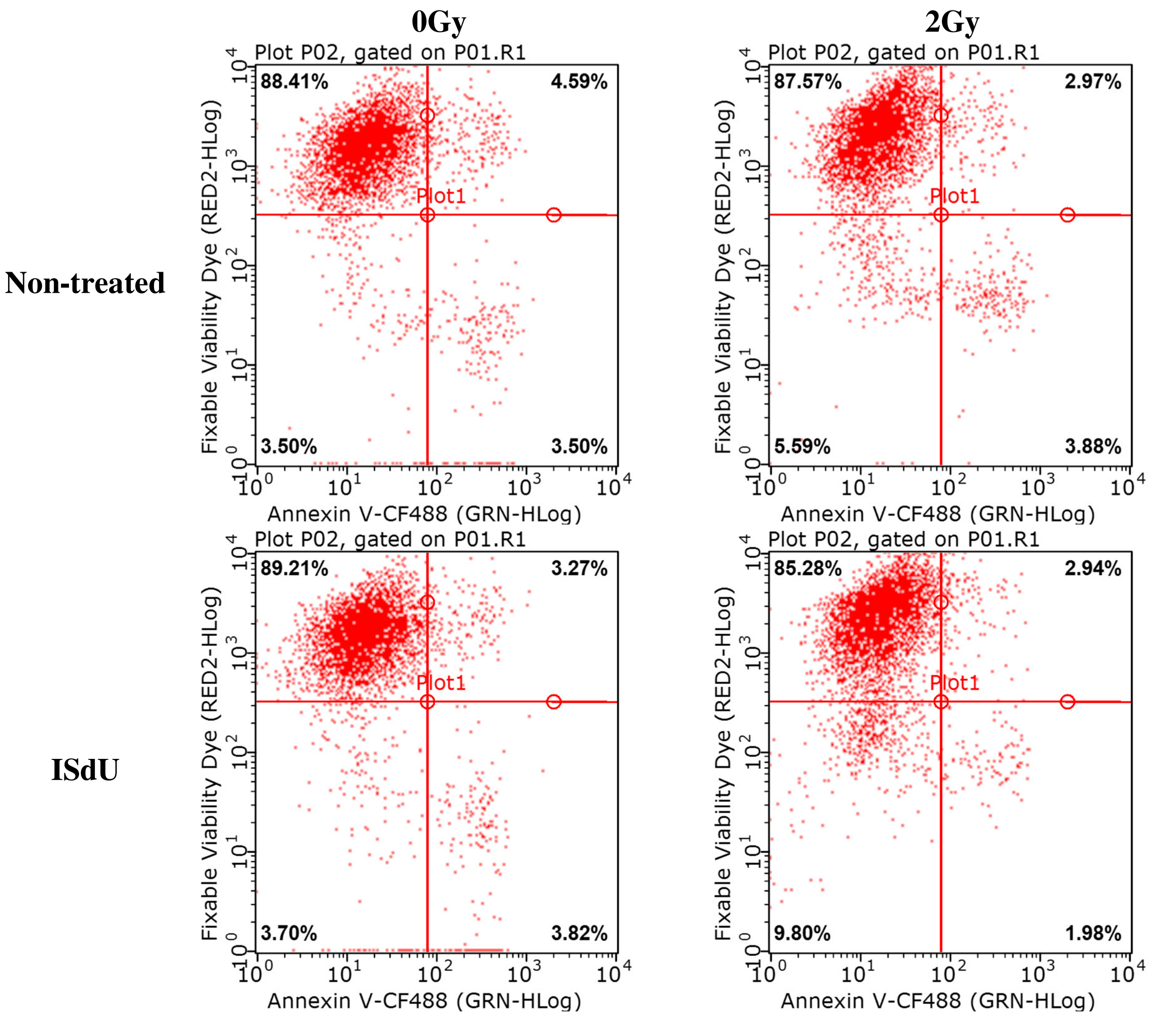

Figure S20. Cytometric analysis of cell death – dot plots provide comparison of MitoSense Dye (early apoptosis) vs. Annexin V (late apoptosis).
